# Reframing Aging-in-the-Right-Place for Housing Insecure Older Adults

**DOI:** 10.1093/geroni/igaa057.2486

**Published:** 2020-12-16

**Authors:** Sarah Canham, Mineko Wada, Stephen Golant

## Abstract

Amidst rising costs of housing and changing personal needs, considerations of the availability of appropriate and accessible housing are becoming increasingly salient for older adults. While it has been widely acknowledged that older adults would prefer to age-in-place, recent reframing of this trend promotes the ideal as aging-in-the-right-place. This symposium will provide an updated understanding of how to support older adults’ ability to age-in-the-right-place, regardless of income or physical, mental, or social status. Presenters include international and interdisciplinary researchers representing perspectives from gerontology, social work, community planning, and health sciences. The symposium will begin with Wada examining resilience scholarship, with a focus on older people who are experiencing homelessness, which has been largely neglected. In the next presentation, Humphries will outline distinct, senior-specific needs and shelter/housing solutions for newly and chronically homeless older adults. Following, Canham will describe promising practices of shelter/housing to support aging-in-the-right-place for older people experiencing homelessness in Montréal, Calgary, and Vancouver identified through an environmental scan. Extending these efforts to an international scale, Mahmood will outline findings from a scoping review of supportive shelter/housing options, supports, and interventions. A final presentation will report on how community development practices implemented by a not-for-profit affordable housing provider promote older tenants’ food security and social support needs. Stephen Golant, a leading expert on housing, geography, and long-term needs on older adults, will discuss implications of these studies for policy and practice for supporting housing insecure older adults while advancing scholarship on aging-in-the-right-place for this marginalized population. Environmental Gerontology Interest Group Sponsored Symposium.

